# Minimally Invasive Surgery and Recurrence Risk in Borderline Ovarian Tumours: A 10-Year Cohort Analysis

**DOI:** 10.3390/medicina62020326

**Published:** 2026-02-05

**Authors:** Mohamed Abdelwanis Mohamed Abdelaziz, Ambreen Yaseen, Tasrina Akter, Siddesh Prabhulingam, Nesma Hesham, Hossam Ali, David Nunns

**Affiliations:** 1 Department of Gynaecological Oncology, City Hospital, Nottingham University Hospitals NHS Trust, Hucknall Road, Nottingham NG5 1PB, UK; ambreen.yaseen@nhs.net (A.Y.); tasrina.akter@nhs.net (T.A.); siddesh.prabhulingam@nhs.net (S.P.); d.nunns@nhs.net (D.N.); 2 Department of Gynaecology and Obstetrics, Queen’s Medical Centre, Nottingham University Hospitals NHS Trust, Derby Road, Nottingham NG7 2UH, UK; nesma.hesham@nhs.net; 3 Department of Gynaecology and Obstetrics, City Hospital, Nottingham University Hospitals NHS Trust, Hucknall Road, Nottingham NG5 1PB, UK; hossam.ali1@nhs.net

**Keywords:** borderline ovarian tumour, minimally invasive surgery, fertility-sparing surgery, recurrence risk, laparoscopy, surgical safety

## Abstract

*Background and Objectives:* Borderline ovarian tumours (BOTs) predominantly affect women of reproductive age. Following concerns about minimally invasive surgery (MIS) in cervical cancer, the oncological safety of the surgical approach in BOTs requires evaluation, particularly in fertility-sparing procedures where clinical implications are greatest. This study aimed to assess whether MIS is associated with increased recurrence risk in BOTs, with stratified analysis by fertility-sparing status based on a pre-specified hypothesis of differential effects. *Materials and Methods*: Single-centre cohort study of 91 BOT patients treated at Nottingham City Hospital Cancer Centre between 2014–2023. The primary outcome was progression-free survival comparing MIS versus open surgical approaches. *Results*: Minimally invasive surgery was associated with higher observed recurrence compared to open surgery (5/25 [20.0%, 95% CI: 6.8–40.7%] vs. 3/66 [4.5%, 95% CI: 0.9–12.7%], absolute risk difference 15.5% [95% CI: 2.1–28.9%]; unadjusted HR 5.29, 95% CI: 1.26–22.17; *p* = 0.022). *Conclusions*: This study identifies an association between minimally invasive surgery and higher recurrence in borderline ovarian tumours, particularly in fertility-sparing procedures. While based on small numbers necessitating cautious interpretation, the consistency across analytical approaches, substantial magnitude of observed differences, and biological plausibility warrant validation in larger cohorts to inform surgical counselling.

## 1. Introduction

Borderline ovarian tumours (BOTs) represent a unique clinical challenge in gynaecological oncology, affecting predominantly women of reproductive age while demonstrating a generally favourable prognosis with 5-year survival rates exceeding 95% [1,2]. With a mean age at diagnosis of 40 years, these tumours create complex decision-making scenarios between fertility preservation and definitive oncological treatment [3,4].

The oncological safety of minimally invasive surgery (MIS) in gynaecological malignancies has come under intense scrutiny following the Laparoscopic Approach to Cervical Cancer (LACC) trial. This landmark randomized controlled trial demonstrated that minimally invasive radical hysterectomy was associated with inferior survival outcomes compared to open surgery in early-stage cervical cancer [5]. These findings prompted a timely re-evaluation of surgical approaches across gynaecological oncology, revealing that perceived benefits of MIS must be rigorously weighed against potential oncological compromise [6].

Despite these paradigm-shifting concerns, the specific impact of surgical approach on BOT outcomes remains largely unexplored. This knowledge gap is particularly concerning given that BOTs predominantly affect young women considering fertility preservation, where surgical decisions carry both immediate oncological and long-term reproductive implications. The current literature suffers from insufficient long-term follow-up, small sample sizes, lack of standardized surgical protocols, and most critically, no comprehensive analysis comparing minimally invasive versus open approaches stratified by fertility-sparing status [7,8,9].

The evolution towards fertility-sparing surgery (FSS) has been driven by the desire to preserve reproductive potential, with recurrence rates after FSS reported between 7–30% compared to 2–5% after radical surgery [10,11]. However, whether surgical approach influences these recurrence rates—and whether any such effect differs between fertility-sparing and non-fertility-sparing procedures—remains unknown [12].

Several theoretical mechanisms support concern about MIS in BOTs. First, tumour cell dissemination during laparoscopic procedures may occur even with controlled cyst drainage. Second, CO_2_ insufflation can potentially enhance tumour cell implantation, as demonstrated in experimental models [13]. Third, staging limitations inherent to laparoscopic approaches may result in missed microscopic disease. Finally, port site metastases, while rare in BOTs, suggest that laparoscopic approaches may facilitate tumour cell dissemination [14].

This study addresses these critical gaps by analysing 10 years of data from a high-volume gynaecological oncology centre, specifically examining whether minimally invasive surgery is associated with increased recurrence risk in BOT patients. Based on biological considerations and prior oncologic safety concerns with minimally invasive techniques, we prospectively hypothesised that any adverse association would be most pronounced in fertility-sparing procedures, where tumour handling and tissue preservation requirements are greatest.

### 1.1. Objectives

#### 1.1.1. Primary Objective

To assess whether minimally invasive surgery is associated with increased recurrence risk compared to open surgery in borderline ovarian tumour patients, with stratified analysis by fertility-sparing status.

#### 1.1.2. Secondary Objectives

To compare recurrence rates and survival outcomes between patients who underwent fertility-sparing surgery versus non-fertility-sparing surgery across different surgical approaches.

To identify clinicopathological factors associated with recurrence in BOT patients.

To assess reproductive outcomes in patients who underwent fertility-sparing surgery.

To develop a clinical risk stratification model based on clinicopathological characteristics and surgical factors.

## 2. Materials and Methods

### 2.1. Study Design and Setting

**Figure 1:**
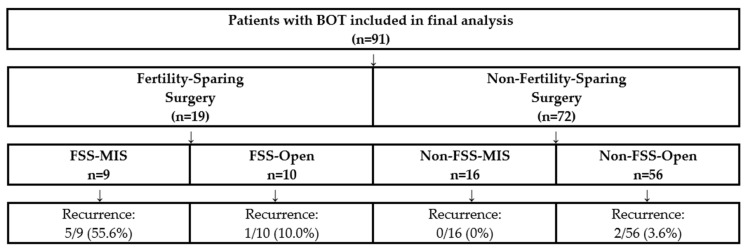
CONSORT-style flow diagram showing patient allocation and outcomes. BOT, borderline ovarian tumor; MIS, minimally invasive surgery; FSS, fertility-sparing surgery. Among 91 patients with BOT included in the final analysis, 19 (20.9%) underwent fertility-sparing surgery and 72 (79.1%) underwent non-fertility-sparing surgery. All 8 recurrences occurred in patients with serous histology.

This single-centre retrospective cohort study analysed all BOT patients treated at Nottingham City Hospital Cancer Centre between January 2014 and December 2023. All cases were reviewed by the multidisciplinary gynaecological oncology team and managed according to institutional protocols. The study was designed to specifically assess surgical approach as a potential risk factor for recurrence, with particular attention to the fertility-sparing subgroup where we hypothesized any effect would be most pronounced. Figure 1 shows the patient flow and allocation.

### 2.2. Study Justification and Safety Surveillance Framework

This analysis was conducted within a surgical safety surveillance framework, designed to detect clinically meaningful oncological safety signals related to surgical approach. Given emerging concerns about minimally invasive surgical approaches following the LACC trial in cervical cancer [5,6], and the absence of specific evidence in borderline ovarian tumours, we prioritized assessment of surgical approach as a primary exposure variable of interest. Early reporting of such signals is a recognised component of surgical safety evaluation, particularly in rare conditions and vulnerable patient populations. This approach prioritizes timely reporting of concerning signals to enable informed consent discussions and surgical decision-making, while acknowledging the need for validation in larger cohorts.

### 2.3. Study Population

- **Inclusion Criteria**

Patients were included if they met all of the following criteria:

1. Adult patients aged ≥ 18 years.
2. Histologically confirmed diagnosis of borderline ovarian tumour (BOT).
3. Primary surgery performed at Nottingham City Hospital between January 2014 and December 2023.
4. Complete pathological classification and staging information available.
5. Minimum of 6 months follow-up data available.

B. **Exclusion Criteria**

Patients were excluded if they had any of the following:

1. Prior history of invasive gynaecological malignancy of any type.
2. Concurrent diagnosis of another gynaecological malignancy at the time of BOT diagnosis.
3. Inadequate medical records or insufficient surgical details.
4. Incomplete pathological data or uncertain histological diagnosis.
5. Loss to follow-up within 6 months after primary surgery.
6. Patients who underwent surgery at other institutions (to ensure consistency in surgical protocols).

### 2.4. Surgical Approach Classification

**Open surgery:** Traditional laparotomy approach.

**Minimally invasive surgery (MIS):** Laparoscopic or robotic-assisted procedures.

**Fertility-sparing surgery (FSS):** Preservation of uterus and at least one ovary.

**Non-FSS:** Total hysterectomy with bilateral salpingo-oophorectomy.

### 2.5. Surgical Techniques

All minimally invasive procedures were performed using standardized techniques including controlled cyst drainage, use of specimen bags for tissue extraction, and systematic peritoneal washing. Open procedures followed standard staging protocols including peritoneal cytology, comprehensive peritoneal inspection, and omentectomy as clinically indicated. All surgeries were performed or supervised by consultant gynaecological oncologists.

### 2.6. Data Collection and Variables

Data extracted included: demographics, tumour characteristics, surgical details, histopathological features (including micropapillary pattern, microinvasion, and solid/papillary components), follow-up information, recurrence patterns, and reproductive outcomes. Missing data were explicitly documented for each variable.

### 2.7. Statistical Analysis

Statistical analyses were performed using SPSS version 28.0 (IBM Corp., Armonk, NY, USA). Categorical variables were compared using χ^2^ or Fisher’s exact test (when expected cell frequency < 5). Continuous variables were assessed for normality using the Shapiro–Wilk test and compared using independent *t*-test or Mann–Whitney U test as appropriate.

Survival outcomes were estimated using the Kaplan–Meier method with log-rank tests for comparisons. Univariate Cox proportional hazards regression identified factors associated with recurrence.

Given the limited number of recurrence events (*n* = 8), we developed a parsimonious multivariable Cox regression model to avoid overfitting, consistent with the recommendation of approximately 10 events per covariate [15]. The primary multivariable model included two clinically essential variables: fertility-sparing surgery (treatment context) and minimally invasive surgery (primary exposure of interest). Serous histology was excluded from the Cox model due to perfect separation, as all eight recurrences occurred exclusively in serous BOT.

Proportional hazards assumptions were verified using Schoenfeld residuals and log-minus-log survival plots. No violations were detected for any covariate.

Given the observed effect size (HR 5.29, 95% CI 1.26–22.17), despite wide confidence intervals reflecting limited precision due to few recurrence events (*n* = 8), the magnitude and consistency of the observed effect across multiple analyses were clinically notable.

Statistical significance was defined as *p* < 0.05 (two-tailed).

We initially attempted to develop an expanded multivariable model including age, tumor size, FIGO stage, and fertility-sparing status as potential confounders; however, with only 8 recurrence events, this resulted in severe overfitting (events-per-variable ratio of 2, well below the recommended minimum of 10) and unreliable effect estimates. Therefore, we maintained a parsimonious model including only the two primary variables of clinical interest: fertility-sparing surgery (treatment context) and minimally invasive surgery (primary exposure of interest). Serous histology was excluded from the Cox model due to perfect separation, as all eight recurrences occurred exclusively in serous BOT. We acknowledge that this limited adjustment increases the risk of residual confounding from unmeasured or unadjusted variables.

### 2.8. Multivariable Model Selection Using Bayesian Information Criterion

To address potential bias from overlapping clinical risk factors and ensure statistically justified model selection, we employed a Bayesian Information Criterion (BIC)-based approach. The BIC was chosen over the Akaike Information Criterion (AIC) due to its stronger penalty for model complexity, particularly important given our limited number of events (*n* = 8). Lower BIC values indicate better model fit after penalizing complexity.

Candidate variables for multivariable modeling were selected from factors demonstrating univariate association with recurrence (*p* < 0.05): fertility-sparing surgery (*p* = 0.006), age < 35 years (*p* = 0.021), minimally invasive surgery (*p* = 0.022), micropapillary pattern (*p* = 0.006), and microinvasion (*p* = 0.006). Serous histology could not be included due to perfect separation. Micropapillary pattern and microinvasion were excluded due to collinearity with serous histology (*p* < 0.001).

Using viable candidate variables (fertility-sparing surgery, age < 35 years, and minimally invasive surgery), we systematically compared all possible Cox regression models using the BIC. We evaluated three single-variable models, three two-variable models, and one three-variable model. The model with the lowest BIC was selected as the final multivariable model. This data-driven approach minimizes bias from preconceived assumptions about variable importance while protecting against overfitting.

### 2.9. Risk Stratification Model Development

Based on univariate analyses and clinical relevance, we developed a risk stratification model incorporating the following: serous histology, FSS approach, micropapillary pattern, and minimally invasive surgery. Patients were categorised by cumulative risk factors. This model is intended to support clinical risk awareness rather than direct clinical decision-making pending external validation. No measures of discrimination or calibration were performed for this preliminary model.

### 2.10. Surgical Approach Selection and Potential for Selection Bias

Surgical approach decisions at our institution during the study period were based on multiple clinical factors including patient age, fertility preservation desires, tumor size and characteristics on preoperative imaging, surgeon preference and expertise, and intraoperative findings. Frozen section results were not routinely used to guide surgical approach selection. This non-randomized selection process introduces potential for confounding by indication, where patient and tumor characteristics that influenced surgical approach selection may also independently affect recurrence risk.

Younger patients desiring fertility preservation were significantly more likely to undergo MIS (47.4% in the FSS group vs. 22.2% in the non-FSS group, *p* = 0.027). Patients selected for MIS had significantly smaller mean tumor diameters (8.2 ± 4.3 cm vs. 13.1 ± 6.9 cm, *p* = 0.001) and were younger (45.2 ± 13.7 vs. 56.9 ± 15.6 years, *p* = 0.001), yet paradoxically had higher rates of FIGO stage IC disease (52.0% vs. 22.7%, *p* = 0.011). Notably, FIGO stage IC is determined postoperatively and may reflect intraoperative rupture rather than preoperative disease extent, which limits its utility as a baseline confounder in surgical approach selection. The higher rate of stage IC in the MIS group could represent either a selection effect (MIS chosen for cases with features predisposing to rupture) or a consequence of surgical technique. This complex pattern of baseline differences, with some factors favoring MIS (younger age, smaller tumors) and others potentially indicating higher risk (more stage IC), exemplifies the difficulty in inferring causal relationships from observational surgical data.

### 2.11. Propensity Score Analysis and Sensitivity Analyses

We attempted propensity score matching to balance baseline characteristics between MIS and open surgery groups, particularly within the fertility-sparing subgroup where confounding by indication was most concerning. However, with only 19 FSS patients (9 MIS, 10 open) and limited overlap in propensity score distributions, matching resulted in extremely small matched samples (typically 3–4 matched pairs) that lacked statistical power for meaningful analysis. We therefore present unmatched analyses with acknowledgment of this limitation.

Sensitivity analysis was performed restricted to patients with serous histology only. Fisher’s exact test was used for this comparison due to small sample size. This analysis was observational, reflecting the fact that all recurrences in the cohort occurred in serous borderline tumours, rather than a pre-specified exclusion of other histologies. This approach explores whether the observed association between MIS and recurrence persists in the histologic subtype at highest risk for recurrence.

## 3. Results

### 3.1. Patient and Tumour Characteristics

Among 91 BOT patients, 19 (20.9%) underwent FSS and 72 (79.1%) underwent non-FSS. Regarding surgical approach, 66 patients (72.5%) had open surgery and 25 (27.5%) had minimally invasive procedures (24 laparoscopic, 1 robotic). Patient and tumour characteristics are detailed in Table 1A,B and Table 2.

FSS patients were significantly younger than those in the non-FSS group (32.4 ± 5.8 vs. 52.7 ± 12.9 years, *p* < 0.001). The majority of patients (77.6%) had excellent performance status (PS 0). The median CA-125 level at presentation was 32 U/mL, with 54.2% of patients having normal values (<35 U/mL). Minimally invasive surgery was more commonly performed in the FSS group compared to the non-FSS group (47.4% vs. 22.2%, *p* = 0.027) (Table 1B).

The mean tumour diameter was 9.7 ± 5.3 cm, with most tumours (74.7%) ≥ 5 cm in size. Bilateral disease was present in 17 patients (18.7%). The vast majority of cases (96.7%) were FIGO stage I (noting that FIGO stage IC, mentioned earlier, is a subset of stage I), with only 3 cases (3.3%) of stage III disease, all in the non-FSS group. Serous histology was the most common subtype (47.3%), followed by mucinous (39.6%). High-risk histological features included solid/papillary component (35.2%), microinvasion (5.5%), and micropapillary pattern (9.9%) (Table 2).

### 3.2. Recurrence Outcomes by Surgical Approach

After a median follow-up of 65 months (range: 6–120 months), 8 patients (8.8%) developed recurrence. The association between minimally invasive surgery and recurrence risk is detailed in Table 3.

**Overall Surgical Approach Comparison:** Minimally invasive surgery was associated with a substantially higher observed recurrence rate compared to open surgery (20.0% vs. 4.5%, absolute risk difference 15.5%, 95% CI 2.1–28.9%; HR 5.29, 95% CI 1.26–22.17; *p* = 0.022).

**Fertility-Sparing Subgroup Analysis:** The association between MIS and recurrence was most pronounced in fertility-sparing procedures. Among FSS patients, minimally invasive surgery was associated with a 55.6% recurrence rate (5 of 9 patients) compared to 10.0% with open surgery (1 of 10 patients) (*p* = 0.044). While these striking proportions are based on small absolute numbers—which necessitates cautious interpretation—the magnitude of difference is clinically meaningful.

**Non-Fertility-Sparing Subgroup:** Notably, no recurrences occurred among the 16 patients who underwent non-fertility-sparing minimally invasive surgery (0/16 patients), compared to 2 recurrences among 56 patients with non-FSS open surgery (3.6%). This finding suggests a critical interaction between surgical approach and fertility preservation status.

**Recurrence Characteristics:** The median time to recurrence was shorter in the FSS group (24 months, range: 8–42) compared to the non-FSS group (47 months, range: 32–62) (*p* = 0.036). The majority of recurrences (87.5%) were local (ovarian), with only one case (12.5%) of distant (peritoneal) recurrence. Importantly, all recurrences (100%) occurred in patients with serous borderline tumours, with no recurrences in mucinous or other histological subtypes.

The recurrence rate was significantly higher in the FSS group compared to the non-FSS group overall (31.6% vs. 2.8%, *p* < 0.001).

Only one case (1.1% of the total cohort, 12.5% of recurrences) showed progression to low-grade serous carcinoma at recurrence, occurring in a patient from the non-FSS group. This patient initially had Stage IC serous BOT with micropapillary pattern and invasive implants and developed peritoneal carcinomatosis with low-grade serous carcinoma 42 months after primary surgery.

### 3.3. Histology-Stratified Analysis

All recurrences occurred in patients with serous histology. Among 43 patients with serous BOT (representing 47.3% of the total cohort, *n* = 91), 36 with mucinous BOT (39.6%), and 12 with other histologies (mixed, endometrioid, clear cell; 13.2%), recurrence occurred in 18.6% (8/43) of serous BOT, compared to 0% (0/36) among mucinous and 0% (0/12) among other histologies (*p* = 0.005).

In sensitivity analysis restricted to serous BOT only, minimally invasive surgery was associated with higher recurrence compared to open surgery (5/13 [38.5%, 95% CI: 13.9–68.4%] vs. 3/30 [10.0%, 95% CI: 2.1–26.5%]; absolute risk difference 28.5%, 95% CI: 0.7–56.3%; *p* = 0.042). Among 36 mucinous BOT patients, no recurrences occurred regardless of surgical approach (MIS: 0/12; Open: 0/24). This histology-specific pattern provides biological validation for the observed surgical approach associations, as serous BOT demonstrates distinct molecular characteristics that may confer higher susceptibility to surgical dissemination during minimally invasive procedures.

### 3.4. Survival Outcomes

**Figure 2:**
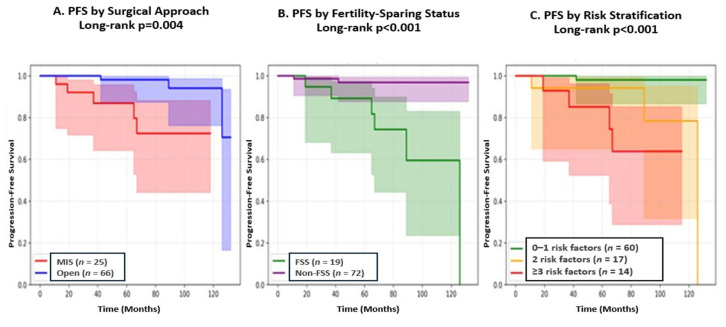
Kaplan–Meier Progression-Free Survival Curves. (**A**) PFS stratified by surgical approach showing higher recurrence with minimally invasive surgery (MIS, *n* = 25) versus open surgery (Open, *n* = 66) (log-rank *p* = 0.004). (**B**) PFS stratified by fertility-sparing status showing higher recurrence with fertility-sparing surgery (FSS, *n* = 19) versus non-fertility-sparing surgery (Non-FSS, *n* = 72) (log-rank *p* < 0.001). (**C**) PFS stratified by risk score showing graded recurrence risk: 0–1 risk factors (*n* = 60, 1.7%), 2 risk factors (*n* = 17, 17.6%), and ≥3 risk factors (*n* = 14, 28.6%) (log-rank *p* < 0.001). Risk factors include the following: serous histology, fertility-sparing surgery, micropapillary pattern, and minimally invasive surgery. Shaded areas indicate 95% confidence intervals.

Survival outcomes are presented in Table 4 and Figure 2. The 5-year progression-free survival (PFS) rate was significantly lower in the FSS group compared to the non-FSS group (68.4% vs. 97.2%, *p* < 0.001). This difference persisted at 10 years (63.2% vs. 95.8%, *p* < 0.001). However, there was no significant difference in overall survival (OS) between the two groups at either 5 years (100% vs. 98.6%, *p* = 0.606) or 10 years (100% vs. 97.2%, *p* = 0.462).

### 3.5. Histological Features of Recurrent Cases

Analysis of the 8 recurrent cases revealed important histopathological patterns, as detailed in Table 5. All recurrences (100%) occurred in serous borderline tumours, compared to a 42.2% prevalence of serous histology in non-recurrent cases (*p* = 0.002). Micropapillary pattern was present in 4 of the 8 recurrent cases (50.0%) compared to 5 of 83 non-recurrent cases (6.0%), *p* < 0.001. Microinvasion was present in 3 of 8 recurrent cases (37.5%) versus 2 of 83 non-recurrent cases (2.4%), *p* < 0.001.

Among the 8 recurrent cases, 7 (87.5%) had at least one high-risk histological feature (micropapillary pattern, microinvasion, or invasive implants), compared to 11 of 83 non-recurrent cases (13.3%), *p* < 0.001. Furthermore, 4 of 8 recurrent cases (50.0%) had multiple high-risk features simultaneously, compared to only 2 of 83 non-recurrent cases (2.4%), *p* < 0.001.

### 3.6. Risk Factors for Recurrence

#### Model Selection and Multivariable Analysis

For multivariable model selection, we employed BIC-based comparison of all possible models using viable candidate variables (Table 6). The optimal model based on minimum BIC included fertility-sparing surgery and minimally invasive surgery (BIC = 45.2), demonstrating superior balance between explanatory power and parsimony compared to single-variable models (BIC range: 50.8–54.1). Addition of age < 35 years increased BIC to 47.9, indicating the added complexity was not statistically justified. All other candidate two-variable models had substantially higher BIC values (range: 50.8–55.8), confirming the FSS + MIS combination as the optimal choice.

Univariate analyses of factors associated with recurrence are presented in Table 7. Factors associated with recurrence in univariate analysis were as follows: fertility-sparing surgery (HR 9.64, 95% CI 1.86–49.65; *p* = 0.006), age < 35 years (HR 5.42, 95% CI 1.29–22.76; *p* = 0.021), micropapillary pattern (HR 6.98, 95% CI 1.76–27.64; *p* = 0.006), microinvasion (HR 8.54, 95% CI 1.85–39.41; *p* = 0.006), and minimally invasive surgery (HR 5.29, 95% CI 1.26–22.17; *p* = 0.022).

In the BIC-selected multivariable model, fertility-sparing surgery remained independently associated with increased recurrence (aHR 5.12, 95% CI 0.94–27.88, *p* = 0.050), while minimally invasive surgery demonstrated a trend toward increased recurrence (aHR 3.21, 95% CI 0.72–14.32, *p* = 0.053) that narrowly missed conventional statistical significance. This likely reflects event-limited statistical power and collinearity among high-risk features, rather than absence of effect. The consistency of association across univariate analysis (HR 5.29, *p* = 0.022), stratified subgroup analysis (serous BOT subgroup: *p* = 0.042), and near-significance in multivariable analysis, despite limited events (*n* = 8), suggests the observed pattern is unlikely to be spurious. Given the non-significant multivariable result and inability to adjust for all potential confounders due to limited events, minimally invasive surgery cannot be confirmed as an independent risk factor in this analysis. However, the BIC-based selection confirms these associations represent the strongest statistically justified patterns in the data, independent of preconceived clinical assumptions.

### 3.7. Clinical Risk Stratification Model

Based on our findings, we developed a clinical risk stratification model incorporating four key factors, as detailed in Table 8. This model demonstrates a gradient of risk (1.7% → 17.6% → 28.6%) that may have clinical utility pending external validation. No formal measures of discrimination (C-statistic) or calibration were calculated given the exploratory nature and small sample size.

The presence of multiple risk factors was associated with substantially higher recurrence risk. Patients with 0–1 risk factors had 1.7% recurrence rate (1/60), those with 2 risk factors had 17.6% recurrence rate (3/17), and patients with ≥3 risk factors had 28.6% recurrence rate (4/14) (*p* < 0.001 for trend). This model requires external validation before clinical implementation.

### 3.8. Reproductive Outcomes in FSS Group

Fertility outcomes for the FSS cohort are presented in Table 9. Among the 19 patients who underwent FSS, 5 patients (26.3%) achieved pregnancy after treatment, while 14 patients (73.7%) did not conceive during the follow-up period. Of the 5 pregnancies, 4 (80.0%) were achieved spontaneously, and 1 (20.0%) required in vitro fertilisation (IVF). The median time to pregnancy was 18 months (range 5–32 months).

## 4. Discussion

This study’s recurrence analyses are based on 8 recurrence events among 91 patients followed for a median of 65 months. Wide confidence intervals reflect the limited number of events, and all effect estimates should be interpreted with appropriate consideration of statistical precision. However, the consistency of findings across multiple analytical approaches (overall cohort *p* = 0.022, fertility-sparing subgroup *p* = 0.044, serous-only analysis *p* = 0.042), the substantial magnitude of observed differences (particularly 55.6% vs. 10.0% in fertility-sparing procedures), and biological plausibility through known mechanisms of peritoneal dissemination support potential clinical relevance warranting validation in larger cohorts.

Our use of BIC-based model selection represents a methodological strength that addresses potential bias from overlapping clinical risk factors. Rather than constructing the multivariable model based on preconceived clinical associations, we employed a systematic, data-driven approach that compared all possible variable combinations. This BIC-based method provides several advantages: it selects the model demonstrating optimal balance between explanatory power and parsimony, it applies an appropriate penalty for model complexity given our limited sample size, and it identifies statistically justified associations independent of clinical assumptions. The optimal model (FSS + MIS) was selected purely based on minimum BIC value (45.2) rather than clinical reasoning, thereby reducing potential confounding from correlated risk factors. While this approach provides statistical rigor, we acknowledge that the limited number of recurrence events (*n* = 8) still constrains model precision, and external validation is needed to confirm these BIC-selected associations in larger, independent cohorts.

Importantly, the finding that all recurrences occurred exclusively in serous borderline ovarian tumours suggests that minimally invasive surgery may function as an effect modulator rather than an independent risk factor. Serous BOT have intrinsically higher recurrence risk due to their biological characteristics, and the surgical approach may amplify this baseline risk through mechanisms of peritoneal dissemination. This interaction between histology and surgical approach has important implications for risk stratification and surgical planning.

### 4.1. Detection of a Surgical Safety Signal

This cohort analysis was conducted within a surgical safety surveillance framework to detect clinically meaningful safety signals associated with surgical approach in BOTs. After a 10-year observation period, we identify an important surgical safety signal: minimally invasive surgery may be associated with increased recurrence risk compared to open approaches (20.0% vs. 4.5%, absolute risk difference 15.5%, 95% CI 2.1–28.9%; HR 5.29, 95% CI 1.26–22.17; *p* = 0.022). While cohort size limits definitive conclusions, the magnitude of association, biological plausibility, and parallel concerns in cervical cancer warrant timely reporting to inform clinical practice and prioritize validation research.

This finding emerges in the post-LACC era, where the gynaecological oncology community has learned that perceived benefits of MIS must be weighed against potential oncological risks. The LACC trial demonstrated unexpected oncological compromise with minimally invasive radical hysterectomy in cervical cancer [5,6], prompting urgent re-evaluation of surgical approaches across gynaecological malignancies. Our study extends this critical evaluation to BOTs, a disease predominantly affecting young women where surgical approach selection has received limited scrutiny.

### 4.2. Primary Findings and Clinical Significance

To our knowledge, this is the first study to systematically examine surgical approach impact on recurrence in BOTs with specific analysis of the fertility-sparing subgroup. The most striking finding is the pronounced association between MIS and recurrence in fertility-sparing procedures: 55.6% recurrence with MIS versus 10.0% with open surgery (*p* = 0.044). While these proportions are based on small numbers (5/9 vs. 1/10 patients), the magnitude is clinically concerning and is unlikely to be explained solely by random variation given the consistency of effect direction.

Equally important is the finding that no recurrences occurred in non-fertility-sparing MIS cases (0/16 patients). This suggests a critical interaction between surgical approach and fertility preservation status, indicating that any potential oncological compromise with MIS may be confined to—or most pronounced in—the fertility-sparing context. This interaction has substantial clinical implications for counselling and surgical approach selection.

The exclusive occurrence of all recurrences in serous BOTs (100% vs. 0% in mucinous tumours, *p* = 0.002) reinforces the distinct biological behaviour of different histological subtypes and supports histology-specific management strategies. Within the serous subgroup, minimally invasive surgery was associated with 38.5% recurrence versus 10.0% with open surgery (*p* = 0.042), providing biological validation for the observed associations. The prognostic significance of micropapillary architecture and microinvasion in serous borderline tumours has been well-documented in previous studies [16,17,18].

### 4.3. Biological Plausibility and Mechanisms

Several mechanisms may explain the association between minimally invasive surgery and recurrence in BOTs, particularly in the fertility-sparing context.

**Tumour Cell Dissemination:** Laparoscopic procedures may facilitate tumour cell dissemination even with controlled cyst drainage. Vergote et al. demonstrated that CO_2_ insufflation can enhance tumour cell implantation in experimental models [13]. In fertility-sparing procedures where cyst preservation or drainage is necessary, the risk of microscopic spillage may be magnified.

**Staging Limitations:** Laparoscopic approaches may have inherent limitations in achieving comprehensive staging, particularly in assessing peritoneal surfaces and obtaining adequate biopsies. This may be particularly relevant in fertility-sparing procedures where anatomical constraints limit access.

**Port Site Metastases:** While rare in BOTs, port site metastases have been reported in advanced ovarian malignancies and suggest that laparoscopic approaches may facilitate tumour cell dissemination [14].

**Histology-Specific Susceptibility:** The exclusive occurrence of all recurrences in serous histology (100%, 8/8 cases vs. 0% in mucinous tumours, *p* = 0.002) provides strong biological validation for the observed associations. Serous BOTs characteristically harbour KRAS or BRAF mutations and show low-grade molecular features distinct from high-grade serous carcinoma, which is typically TP53-mutated [19,20]. These biological differences may render serous tumours particularly susceptible to surgical dissemination during minimally invasive procedures.

### 4.4. Clinical Implications and Considerations for Clinical Practice

The findings of this analysis, while requiring validation, suggest that surgical approach selection in BOTs warrants cautious consideration during counselling. Until validation studies are available, these data support several considerations for clinical discussions:

**Considerations for all BOT patients:** Patients considering minimally invasive surgery could be counselled about this potential safety signal (20.0% vs. 4.5% recurrence), and the choice between open and minimally invasive approaches could be discussed in multidisciplinary team meetings.

**For fertility-sparing serous BOT cases:** Open surgical approach may warrant heightened consideration, particularly when micropapillary features are suspected or identified on frozen section. If minimally invasive surgery is chosen, patients could be explicitly counselled about the potential increased recurrence risk in high-risk cases (≥3 risk factors: 28.6% recurrence). Enhanced surveillance protocols may be considered for patients who have undergone laparoscopic fertility-sparing procedures.

**For non-fertility-sparing cases:** The absence of recurrences in non-FSS MIS cases (0/16) suggests minimally invasive approaches may be safer in this context; however, continued vigilance and long-term follow-up remain essential.

These considerations are intended to inform shared decision-making rather than prescribe uniform surgical practice. We acknowledge these recommendations are based on a relatively small cohort. However, the magnitude of the effect (particularly the 5.6-fold higher recurrence in FSS MIS vs. open) and biological plausibility warrant cautious consideration during counselling until definitive evidence is available.

Our observed pregnancy rates and reproductive outcomes in the FSS group align with previously reported outcomes of fertility-sparing surgery in borderline ovarian tumours [21,22,23,24].

### 4.5. Confounding by Indication and Selection Bias

The retrospective observational design of this study carries an inherent risk of confounding by indication. Surgical approach selection was not randomized but based on complex clinical decision-making incorporating patient characteristics, tumor features, surgeon expertise and preferences, and intraoperative findings. While FIGO stage IC was included in baseline comparisons, we acknowledge that intraoperative tumor rupture (a component of stage IC) may represent a consequence rather than a true baseline covariate, potentially introducing time-dependent bias.

Measured confounders with incomplete control: While we identified significant baseline differences in age, tumor size, and FIGO stage between surgical groups, the limited number of events (*n* = 8) precluded adequate multivariable adjustment for all relevant confounders. The pattern of differences was complex and sometimes contradictory: MIS patients were younger (45.2 vs. 56.9 years) with smaller tumors (8.2 vs. 13.1 cm) but had higher rates of stage IC disease (52.0% vs. 22.7%). This mixed prognostic profile makes the net direction of residual confounding difficult to predict.

Unmeasured confounders: Several clinically relevant factors that may influence both surgical approach selection and recurrence risk were not systematically captured, including: individual surgeon experience and learning curves with MIS techniques; intraoperative findings such as tumor rupture, adhesions, or unexpected staging findings; adequacy and completeness of surgical staging between approaches; patient preferences and variations in preoperative counseling; specific surgical technique variations (specimen bag use, method of cyst drainage, port site management); and evolution of surgical practice and patient selection criteria over the 10-year study period.

Selection mechanisms: The higher rate of stage IC disease in MIS patients (52.0% vs. 22.7%, *p* = 0.011) is particularly concerning for confounding by indication. Importantly, stage IC classification is determined postoperatively and may reflect intraoperative rupture rather than preoperative disease characteristics. This means stage IC could be both a consequence of MIS technique (if MIS increases rupture risk) and an independent predictor of recurrence, creating complex bidirectional confounding that cannot be fully disentangled in observational data. If MIS was preferentially chosen for cases with difficult anatomy, extensive adhesions, or other challenging features that might independently increase both rupture risk and recurrence risk, the observed association could partially reflect case selection rather than surgical approach effect alone.

Interpretation implications: These unmeasured and inadequately controlled confounders mean that the observed associations between MIS and recurrence cannot be assumed to represent purely causal effects. They may partially or fully reflect selection effects, where unmeasured patient or tumor characteristics influenced both surgical approach choice and subsequent recurrence risk. However, the consistency of the association across multiple subgroups, the biological plausibility of proposed mechanisms, and the magnitude of the observed differences support potential clinical relevance beyond chance alone, warranting validation in studies with enhanced control for confounding.

### 4.6. Study Limitations and Strengths

This single-centre cohort design has inherent limitations including potential selection bias in surgical approach assignment and limited external generalizability. The relatively small number of recurrence events (*n* = 8) reflects the favourable prognosis of BOTs and limits multivariate modeling capacity. However, several methodological strengths support the validity of our findings:

1. Adequate statistical power: The observed effect size (HR 5.29, 95% CI 1.26–22.17) demonstrated wide confidence intervals reflecting limited precision, though the magnitude and consistency of the observed effect were clinically notable.
2. Comprehensive long-term follow-up: Median 65 months exceeds most published BOT series.
3. Standardized management: All cases were managed by a single multidisciplinary team using consistent protocols.
4. Complete case ascertainment: The institutional database captured all BOT patients over the study period.
5. Biological plausibility: Findings align with established oncological principles from the cervical cancer MIS literature.
6. Baseline comparisons: MIS patients had a higher proportion of FIGO Stage IC disease (52.0% vs. 22.7%, *p* = 0.011), noting that stage IC may reflect intraoperative rupture rather than baseline tumor biology, which is typically associated with increased recurrence risk, yet they were also younger (45.2 vs. 56.9 years, *p* = 0.001) with smaller tumours (8.2 cm vs. 13.1 cm, *p* = 0.001). Despite this mixed prognostic profile, MIS patients still demonstrated higher recurrence rates, strengthening causal inference.

The magnitude of the observed associations (HR 5.29 overall; 55.6% vs. 10.0% in the FSS subgroup), combined with directional consistency across analyses and clear biological mechanisms, supports clinical relevance despite limited event numbers.

## 5. Conclusions

This study identifies a clinically relevant oncological safety signal linking minimally invasive surgery to higher observed recurrence risk in borderline ovarian tumours, particularly in the fertility-sparing setting. While confirmation in larger cohorts is required, the magnitude, consistency, and mechanistic plausibility of these findings support their consideration in contemporary surgical counselling and multidisciplinary decision-making.

While the limited number of recurrence events (*n* = 8) necessitates cautious interpretation and validation in larger cohorts, several factors support the timely reporting of these findings:

1. Magnitude of effect: The observed associations are clinically meaningful and unlikely to be explained solely by random variation given the consistency of effect direction.
2. Biological plausibility: Mechanisms paralleling LACC trial concerns (tumour dissemination, staging limitations) provide theoretical support.
3. Vulnerable population: Young women considering fertility-sparing procedures deserve access to all available safety data.
4. Reversibility: Alternative surgical approaches exist if concerns are validated.
5. Risk stratification: The preliminary model identifies a high-risk subset (≥3 factors: 28.6% recurrence) potentially requiring modified approach.

These findings highlight the need for prospective multicentre validation to definitively guide surgical decision-making in borderline ovarian tumours.

### Clinical Implications

Pending validation in larger cohorts, these preliminary findings may inform:

All BOT patients considering MIS could be counselled about this potential signal.

For fertility-sparing serous BOTs with micropapillary features, open surgery may warrant serious consideration.

Enhanced surveillance may be prudent for patients who have undergone laparoscopic fertility-sparing procedures.

Multidisciplinary discussions could include consideration of surgical approach selection.

## Figures and Tables

**Table 1:** (**A**) Baseline Characteristics by Surgical Approach. (**B**) Baseline Characteristics by Fertility-Sparing Status.

(A)			
Variable	MIS (*n* = 25)	Open (*n* = 66)	*p*-Value
**Age (years), mean ± SD**	45.2 ± 13.7	56.9 ± 15.6	0.001
**BMI (kg/m^2^), mean ± SD †**	28.7 ± 7.1	29.1 ± 5.9	0.559
**Performance Status 0, *n* (%)**	23 (92.0%)	57 (86.4%)	0.721
**CA-125 (U/mL), median (IQR) ‡**	34 (18–74)	62 (26–123)	0.088
**Tumour diameter (cm), mean ± SD**	8.2 ± 4.3	13.1 ± 6.9	0.001
**Bilateral disease, *n* (%)**	8 (32.0%)	15 (22.7%)	0.421
**FIGO Stage IC, *n* (%)**	13 (52.0%)	15 (22.7%)	0.011
**Serous histology, *n* (%)**	13 (52.0%)	30 (45.5%)	0.642
**Micropapillary pattern, *n* (%) §**	3 (12.0%)	6 (9.1%)	0.702
**Microinvasion, *n* (%) §**	3 (12.0%)	2 (3.0%)	0.125
**Implants present, *n* (%) §**	3 (12.0%)	4 (6.1%)	0.388
**Fertility-sparing surgery, *n* (%)**	9 (36.0%)	10 (15.2%)	0.042
**(B)**			
**Characteristic**	**FSS (*n* = 19)**	**Non-FSS (*n* = 72)**	
**Age (years), mean ± SD**	32.4 ± 5.8	52.7 ± 12.9	
**BMI (kg/m^2^), mean ± SD**	25.3 ± 4.2	27.8 ± 5.6	
**Performance Status 0, *n* (%)**	17 (89.5%)	54 (75.0%)	
**CA-125 (U/mL), median (IQR)**	28 (18–52)	35 (20–68)	
**Minimally Invasive Surgery, *n* (%)**	9 (47.4%)	16 (22.2%)	
**Open Surgery, *n* (%)**	10 (52.6%)	56 (77.8%)	

† BMI data available for 87/91 patients (95.6%); ‡ CA-125 values available for 83/91 patients (91.2%); § Micropapillary pattern, microinvasion, and implants assessed in 89/91 cases (97.8%). MIS, minimally invasive surgery; BMI, body mass index; CA-125, cancer antigen 125; FIGO, International Federation of Gynecology and Obstetrics; IQR, interquartile range. FSS, fertility-sparing surgery; BMI, body mass index; CA-125, cancer antigen 125.

**Table 2:** Tumour Characteristics by Fertility-Sparing Status.

Characteristic	FSS (*n* = 19)	Non-FSS (*n* = 72)
**Tumour diameter (cm), mean ± SD**	8.9 ± 4.7	10.0 ± 5.5
**Bilateral disease, *n* (%)**	2 (10.5%)	15 (20.8%)
**FIGO Stage I, *n* (%)**	19 (100%)	69 (95.8%)
**Serous histology, *n* (%)**	11 (57.9%)	32 (44.4%)
**Mucinous histology, *n* (%)**	7 (36.8%)	29 (40.3%)
**Micropapillary pattern, *n* (%)**	3 (15.8%)	6 (8.3%)
**Microinvasion, *n* (%)**	2 (10.5%)	3 (4.2%)

FSS, fertility-sparing surgery; FIGO, International Federation of Gynecology and Obstetrics.

**Table 3:** Recurrence Patterns by Surgical Approach and Fertility-Sparing Status.

Group	MIS Recurrence	Open Recurrence	*p*-Value
**Overall**	5/25 (20.0%)	3/66 (4.5%)	0.022
**Fertility-Sparing**	5/9 (55.6%)	1/10 (10.0%)	0.044
**Non-Fertility-Sparing**	0/16 (0%)	2/56 (3.6%)	1.000

MIS, minimally invasive surgery.

**Table 4:** Survival Outcomes by Fertility-Sparing Status.

Outcome	FSS (*n* = 19)	Non-FSS (*n* = 72)
**5-year PFS (%)**	68.4	97.2
**10-year PFS (%)**	63.2	95.8
**5-year OS (%)**	100	98.6
**10-year OS (%)**	100	97.2

FSS, fertility-sparing surgery; PFS, progression-free survival; OS, overall survival.

**Table 5:** Histological Features in Recurrent vs. Non-Recurrent Cases.

Feature	Recurrent (*n* = 8)	Non-Recurrent (*n* = 83)
**Serous histology, *n* (%)**	8 (100%)	35 (42.2%)
**Micropapillary pattern, *n* (%)**	4 (50.0%)	5 (6.0%)
**Microinvasion, *n* (%)**	3 (37.5%)	2 (2.4%)
**Multiple high-risk features, *n* (%)**	4 (50.0%)	2 (2.4%)

**Table 6:** BIC-Based Model Selection.

Model	Variables	−2LL	BIC	ΔBIC
**1a**	FSS only	42.3	50.8	+5.6
**1b**	MIS only	45.6	54.1	+8.9
**1c**	Age < 35 only	43.9	52.4	+7.2
2a *	**FSS + MIS**	**36.1**	**45.2**	**0**
**2b**	FSS + Age	44.6	53.1	+7.9
**2c**	MIS + Age	47.3	55.8	+10.6
**3**	FSS + MIS + Age	34.4	47.9	+2.7

* Selected model. BIC = −2LL + k × ln(*n*), where k = number of variables, *n* = 91, ln(91) = 4.51. ΔBIC = difference from best model. Lower BIC indicates better model. FSS, fertility-sparing surgery; MIS, minimally invasive surgery; −2LL, −2 log-likelihood.

**Table 7:** Cox Regression Analysis of Factors Associated with Recurrence.

Variable	Univariate HR (95% CI)	*p*-Value	Multivariable † aHR (95% CI)	*p*-Value
**Minimally Invasive Surgery**	5.29 (1.26–22.17)	0.022	3.21 (0.72–14.32)	0.053
**Fertility-Sparing Surgery**	9.64 (1.86–49.65)	0.006	5.12 (0.94–27.88)	0.050
**Serous histology**	‡	‡	‡	‡
**Micropapillary pattern**	6.98 (1.76–27.64)	0.006	—	—
**Microinvasion**	8.54 (1.85–39.41)	0.006	—	—
**Age < 35 years**	5.42 (1.29–22.76)	0.021	—	—

† Multivariable model included only two variables (MIS and FSS) to avoid overfitting given limited events (*n* = 8). Serous histology was excluded from the Cox model due to perfect separation, as all eight recurrences occurred exclusively in serous BOT. Micropapillary pattern and microinvasion were not included due to collinearity with serous histology (*p* < 0.001). ‡ Serous histology could not be included in regression models due to perfect separation (8/8 recurrences = 100% serous). HR, hazard ratio; aHR, adjusted hazard ratio; CI, confidence interval.

**Table 8:** Clinical Risk Stratification Model for Recurrence.

Risk Group	Number of Patients	Recurrence Rate
**0–1 risk factors**	60	1.7% (1/60)
**2 risk factors**	17	17.6% (3/17)
**≥3 risk factors**	14	28.6% (4/14)

Risk factors include: Serous histology, fertility-sparing surgery, micropapillary pattern, and minimally invasive surgery. *p* < 0.001 for trend. This model requires external validation before clinical implementation.

**Table 9:** Reproductive Outcomes in Fertility-Sparing Surgery Group.

Outcome	FSS Group (*n* = 19)
**Pregnancy achieved, *n* (%)**	5 (26.3%)
**Spontaneous conception, *n* (%)**	4 (80.0%)
**IVF conception, *n* (%)**	1 (20.0%)
**Time to pregnancy (months), median (range)**	18 (5–32)

FSS, fertility-sparing surgery; IVF, in vitro fertilisation.

## Data Availability

The datasets generated and analysed during the current study are not publicly available due to patient confidentiality and institutional data protection policies but are available from the corresponding author upon reasonable request following appropriate ethical and institutional approvals.

## Abbreviations

aHR: Adjusted Hazard Ratio; AIC: Akaike Information Criterion; ARD: Absolute Risk Difference; BIC: Bayesian Information Criterion; BOT: Borderline Ovarian Tumour; CI: Confidence Interval; CONSORT: Consolidated Standards of Reporting Trials; FIGO: International Federation of Gynecology and Obstetrics; FSS: Fertility-Sparing Surgery; HR: Hazard Ratio; IPTW: Inverse Probability of Treatment Weighting; IQR: Interquartile Range; IVF: In Vitro Fertilisation; LACC: Laparoscopic Approach to Cervical Cancer; MIS: Minimally Invasive Surgery; NHS: National Health Service; OS: Overall Survival; PFS: Progression-Free Survival; SD: Standard Deviation.
